# Differential effects of growth restriction and immaturity on predicted psychomotor development at 4 years of age in preterm infants

**DOI:** 10.1016/j.xagr.2023.100305

**Published:** 2024-01-09

**Authors:** Arne Jensen, Niels Rochow, Manfred Voigt, Gerhard Neuhäuser

**Affiliations:** 1 Campus Clinic Gynecology, Ruhr-University Bochum, Germany (Dr Jensen); 2 Department of Pediatrics, Paracelsus Medical University, Nuremberg, Germany (Dr Rochow); 3 Department of Pediatrics, University Medicine Rostock, Rostock, Germany (Dr Rochow); 4 DEUZWEG German Center for Growth, Development and Health Promotion in Childhood and Adolescence, Berlin, Germany (Drs Rochow and Voigt); 5 Institute for Perinatal Growth Research, Sievershagen, Germany (Dr Voigt); 6 Department of Paediatric Neurology, University of Giessen, Germany (Dr Neuhäuser).

**Keywords:** Apgar score, asymmetric growth restriction, birth asphyxia, birthweight percentiles, cerebral palsy, disability, infantile brain injury, intelligence quotient, intrauterine growth restriction, Porteus Maze test, neurologic examination optimality score, preterm birth, psychopathology in children, risk stratification, school performance

## Abstract

**BACKGROUND:**

Fetal growth restriction and immaturity are associated with poor neurocognitive development and child psychopathology affecting educational success at school and beyond. However, the differential effects of either obstetrical risk factor on predicted psychomotor development have not yet been deciphered.

**OBJECTIVE:**

This study aimed to separately study the impact of growth restriction and that of immaturity on predicted psychomotor development at the preschool age of 4.3 (standard deviation, 0.8) years using birthweight percentiles in a prospective cohort of preterm infants born at ≤37^+6/7^ weeks of gestation. Differences between small for gestational age newborns with intrauterine growth restriction and those without were described. We examined *predicted* total psychomotor development score, *predicted* developmental disability index, *calculated* morphometric vitality index, and *predicted* intelligence quotient, Porteus Maze test score, and neurologic examination optimality score in 854 preterm infants from a large prospective screening cohort (cranial ultrasound screening, n=5,301).

**STUDY DESIGN:**

This was a prospective cranial ultrasound screening study with a single-center cohort observational design (data collection done from 1984–1988, analysis done in 2022). The study included 5,301 live-born infants, of whom 854 (16.1%) were preterm infants (≤37^+6/7^ weeks’ gestation), and was conducted on the day of discharge of the mother at 5 to 8 days postpartum from a level 3 perinatal center. Predicted psychomotor development, as assessed by the *predicted* total psychomotor development score, *predicted* developmental disability index, *calculated* morphometric vitality index, *predicted* intelligence quotient, Porteus Maze test score, and neurologic examination optimality score were calculated. We related psychomotor development indices and measures to gestational age in 3 groups of birthweight percentiles (ie, 10%, 50%, and 90% for small, appropriate, and large for gestational age newborns, respectively) using linear regression analysis, analysis of variance, multivariate analysis of variance, and *t* test procedures.

**RESULTS:**

The key result of our study is the observation that in preterm infants born at ≤37^+6/7^ weeks of gestation, growth restriction as compared with immaturity is the prime risk factor for impairment of overall *predicted* psychomotor development, intelligence quotient, Porteus Maze test results, and neurologic examination optimality score at the preschool age of 4.3 (standard deviation, 0.8) years (*P*<.001). This is particularly true for intrauterine growth restriction. These detrimental effects of growth restriction become more prominent with decreasing gestational age (*P*<.001). As expected, growth restriction in preterm infants born at ≤37^+6/7^ weeks of gestation was associated with a number of obstetrical risk factors, including hypertension in pregnancy (*P*<.001), multiple pregnancy (*P*<.001), pathologic cardiotocography (*P*=.001), and low pH (*P*=.007), increased pCO2 (*P*=.009), and poor pO2 (*P*<.001) in umbilical arterial blood. Of note, there were no differences in cerebral hemorrhage or white matter damage among small, appropriate, and large for gestational age birthweight percentile groups, suggesting an independent mechanism of brain injury caused by preterm growth restriction resulting in poor psychomotor development.

**CONCLUSION:**

Compared with immaturity, growth restriction in preterm infants has more intense detrimental effects on psychomotor development, necessitating improved risk stratification. This finding has implications for clinical management, parental consultation, and early intervention strategies to improve school performance, educational success, and mental health in children. The mechanisms of brain injury specific to growth restriction in preterm infants require further elucidation.

**AJOG Global Reports at a Glance**

**Why was this study conducted?**

We explored the differential effects of growth restriction and immaturity on *predicted* psychomotor development to improve risk stratification, clinical management, early intervention strategies, preschool support, and mental health in children.

**Key findings**

Compared with immaturity, the detrimental effects of growth restriction on *predicted* psychomotor development at 4 years of age in preterm infants born at ≤37^+6/7^ weeks of gestation are much more intense and increase with decreasing gestational age.

**What does this add to what is known?**

Apparently independently of cerebral hemorrhage and/or white matter damage, growth-restricted preterm infants fare far worse in *predicted* psychomotor development than solely immature infants at the same gestational age. This suggests specific brain damage occurring during chronic hypoxemia and malnutrition, circulatory centralization, and brain-sparing requiring timely risk stratification to improve management in clinical obstetrics, parental consultation, and early intervention strategies to prevent harm.

## Introduction

In newborns, obstetrical risk factors such as growth restriction and immaturity are associated with poor neurocognitive development and child psychopathology that affect school performance and educational success.^1^, ^2^, ^3^, ^4^, ^5^, ^6^, ^7^, ^8^, ^9^, ^10^, ^11^, ^12^, ^13^, ^14^ However, the differential effects of either obstetrical risk factor on *predicted* psychomotor development are not well understood.^3^ We therefore studied the differential impact of both growth restriction and immaturity on *predicted* psychomotor development at the preschool age of 4.3 (standard deviation [SD], 0.8) years using birthweight percentiles in preterm infants born at ≤37^+6/7^ weeks of gestation derived from a prospective cranial ultrasound database that has been validated for the prediction of psychomotor development in previous accounts.^1^, ^2^, ^3^ Distinguishing the detrimental effects of general growth restriction, intrauterine growth restriction (IUGR) in particular, and immaturity on psychomotor development in preterm infants may be important for clinical management in obstetrics, parental consultation, and the orchestration of appropriate early intervention strategies to improve school performance and reduce child psychopathology.^5^^,^^7^^,^^8^^,^^13^, ^14^, ^15^, ^16^, ^17^, ^18^ Furthermore, there is evidence that the mechanisms of growth restriction causing poor psychomotor development may be different from those elicited by brain injury caused by cerebral hemorrhage or white matter damage.^19^, ^20^, ^21^, ^22^

## Materials and Methods

Methodologic details for the prediction of psychomotor development at preschool age have been described elsewhere.^1^, ^2^, ^3^ Briefly, a prospective cranial ultrasound screening study was conducted from 1984 to 1988 at a level III perinatal center at the University of Giessen, Germany. The study included 5,301 live-born infants (after excluding 498 [8.6%] who were discharged early, ie, at ≤4 days), of whom 854 (16.1%) were preterm infants (≤37^+6/7^ weeks’ gestation), and was conducted on the day of discharge of the mother at 5 to 8 days postpartum.^2^^,^^3^ In a previous study (1982–1986) from the same center, both cranial ultrasound screening results after birth and psychomotor development were determined in 137 (2.4%) children at the preschool age of 4.3 (SD, 0.8) years. The study had a matched-pair design, strictly controlling for confounders, for example, sex, socioeconomic status, maternal education, and brain damage.^1^^,^^2^^,^^23^ Intelligence quotient (IQ), Porteus Maze test (PMT) score (adapted by Kramer,^24^ 1985), and neurologic examination optimality score (NOS) were *measured* (*m*), and an average composite total psychomotor development score (TPMDS) for overall psychomotor development was formed (*m*TPMDS=[zIQ+zPMT result+zNOS]/3).^24^, ^25^, ^26^, ^27^ These psychomotor development data were extrapolated to the whole ultrasound screening cohort (n=5,301) as follows. The *measured* psychomotor development testing results, as assessed by the TPMDS, were used to generate a *prediction* model with *measured* TPMDS as the dependent variable by stepwise multiple regression analysis (*p*TPMDS=−17.87+0.00043 × weight−0.501 × white matter damage (WMD)_present+2.278 × pH_umb.art+0.177 × mode of delivery; *r*=0.637; n=129; *P*<.001), which correlated well with the *measured* results (*r*=0.598; n=130; *P*<.001) and hence was used for extrapolation (n=5,301).^3^ Secondly, using *predicted* (*p*) IQ (*p*IQ=−153.61−1.545 × Brain bodyweight ratio (BBR)+43.987 × pH; *r*=0.459; n =131; *P*<.001), *predicted* PMT (*p*PMT=541.20+0.14 × weight+23.176 × IUGR−12.064 × Peri-/intraventricular hemorrhage (PIVH)-1+2_present+67.606 × pH; *r*=0.516; n=133; *P*<.001), and *predicted* NOS (*p*zNOS=−14.03+0.30 × weight/length-ratio−0.623 × WMD_present–0.353 × PIVH-1+2_present+1.683 × pH+0.326 × mode of delivery–0.366 × pathologic cardiotocography; *r*=0.605; n=132; *P*<.001), a *predicted* developmental disability index (DDI) was formed on the basis of various degrees of infantile brain dysfunction (IBD) and cerebral palsy (CP), as described elsewhere.^1^ The *predicted* DDI (*p*DDI) was derived by stepwise multiple regression including all growth and obstetrical risk variables and cranial ultrasound results at birth using the grouped results of controls, IBD-0, IBD-1, IBD-2, and CP as dependent variables to *predict* the degree of IBD and CP (*p*DDI=25.218−0.00057 × weight (g)+0.999 × WMD_present−0.141 × Apgar_10−0.320 × mode of delivery−2.934 × pH_umb.art.; *r*=0.642; n=130; *P*<.001). Again, the *predicted* index *p*DDI correlated well with the *measured* TPMDS (*p*DDI=0.747−0.603 × *m*TPMDS; *r*=0.598; n=130; *P*<.001).^1^^,^^3^ Finally*,* the *calculated* (*c*) morphometric vitality index (MVI) (*c*MVI=[zWeight+zLength+zHeadCircumference+zWeight/length+zApgar_10]/5) was obtained from all 5,301 newborns, and it correlated well with the *predicted* TPMDS (z*p*TPMDS=0.166+0.702 × *c*MVI; *r*=0.844; n=5191; *P*<.001).^1^^,^^3^

To describe the distinct effects of both growth restriction and immaturity on neurocognition, we related psychomotor development indices and measures to gestational age defined by date of last menstruation in 3 groups of birthweight percentiles (ie, 10% [small for gestational age (SGA)], 50% [appropriate for gestational age (AGA)], and 90% [large for gestational age (LGA)]). This report follows the STROBE (Strengthening the Reporting of Observational Studies in Epidemiology) reporting guideline for observational studies.

### Statistical analysis

Results are presented as means and SD. The a priori level of significance for rejecting the null hypothesis was set at a 2-tailed alpha level of <0.05. We evaluated growth restriction and immaturity at birth in relation to z-score transformed (z) *predicted* psychomotor development indices and measures using parametric and nonparametric statistical procedures, analysis of variance, multivariate analysis of variance, and analysis of covariance, as appropriate. To account for multiple comparisons, the Games–Howell test was used. All procedures were performed using the IBM SPSS Statistics, Version 28.0 (IBM Corp, Armonk, NY) statistical software. Deviations from the total number of participants are due to missing values.

## Results

A total of 854 (16.1%) preterm infants born at ≤37^+6/7^ weeks (51.8% male) underwent cranial ultrasound screening (including twins). No sex-related differences were found in the overall rate of cerebral hemorrhage, white matter damage, Apgar scores at 1, 5, and 10 minutes, or umbilical artery pH.

The 854 preterm infants had the following characteristics: mean gestational age from last menstrual period of 34.5 weeks (SD, 2.9; range, 24–37), weight of 2224 g (SD, 721; range, 350–4800), total body length of 45.2 cm (SD, 5.0; range, 25–57), head circumference of 31.4 cm (SD, 3.1; range, 21–39), Apgar score at 10 minutes ≤9 (341/854; range, 3–9), and umbilical arterial pH of 7.28 (SD, 0.09; range, 6.65–7.46). Mean z*p*TPMDS was −0.77 (SD, 0.9; range, −4.0 to 2.1). Mean birthweight, length, and head circumference percentiles were 33.7 (SD, 26.6; range, 0.0–100), 37.2 (SD, 27.0; range, 0.0–100), and 37.4 (SD, 27.4; range, 0.0–100), respectively. For further analysis, 3 groups of birthweight percentiles (ie, 10% [SGA], 50% [AGA], and 90% [LGA]) were used (n=302). The mean birthweight percentile was 3.75 (SD, 3.02; range, 0.00–9.97) in the SGA group, 54.35 (SD, 2.85; range, 50.06–59.87) in the AGA group, and 95.10 (SD, 3.23; range, 90.18–100) in the LGA group. The group-related characteristics and their birth and obstetrical risk factors are given in Tables 1 and 2.

**Table 1:** Birth-related risk factors and psychomotor development indices and measures in preterm infants (24–37 weeks’ gestation; n=302) derived from a prospective cranial ultrasound screening database (n=5,301) in 3 birthweight percentile groups (ie, small [10%], appropriate [50%], and large [90%] for gestational age)

						95% confidence interval					
Variable	Percentile (%)	N	Mean	Standard deviation	Standard error	Lower limit	Upper limit	Minimum	Maximum	Welch test*P* value	Games–Howell*P* value
Sex	10	213	1.52	0.50	0.03	1.45	1.58	1.00	2.00		
	50	61	1.48	0.50	0.06	1.35	1.60	1.00	2.00		
	90	28	1.54	0.51	0.10	1.34	1.73	1.00	2.00		
	Total	302	1.51	0.50	0.03	1.45	1.57	1.00	2.00	.823	
PIVH present	10	213	0.20	0.40	0.03	0.14	0.25	0.00	1.00		
	50	61	0.18	0.39	0.05	0.08	0.28	0.00	1.00		
	90	28	0.18	0.39	0.07	0.03	0.33	0.00	1.00		
	Total	302	0.19	0.39	0.02	0.15	0.24	0.00	1.00	.94	
WMD present	10	213	0.17	0.38	0.03	0.12	0.23	0.00	1.00		
	50	61	0.16	0.37	0.05	0.07	0.26	0.00	1.00		
	90	28	0.14	0.36	0.07	0.00	0.28	0.00	1.00		
	Total	302	0.17	0.38	0.02	0.13	0.21	0.00	1.00	.908	
Apgar 1 min	10	212	7.00	2.11	0.15	6.71	7.29	1.00	10.00		.02
	50	61	7.07	2.13	0.27	6.52	7.61	1.00	9.00		
	90	28	7.82	1.39	0.26	7.28	8.36	4.00	9.00		
	Total	301	7.09	2.07	0.12	6.86	7.32	1.00	10.00	.027	
Apgar 5 min	10	212	8.68	1.37	0.09	8.50	8.87	3.00	10.00		
	50	61	8.72	1.28	0.16	8.39	9.05	4.00	10.00		
	90	28	9.07	1.44	0.27	8.51	9.63	4.00	10.00		
	Total	301	8.73	1.36	0.08	8.57	8.88	3.00	10.00	.412	
Apgar 10 min	10	210	9.29	0.93	0.06	9.16	9.42	6.00	10.00		
	50	59	9.34	0.88	0.11	9.11	9.57	7.00	10.00		
	90	27	9.52	0.94	0.18	9.15	9.89	7.00	10.00		
	Total	296	9.32	0.92	0.05	9.22	9.43	6.00	10.00	.491	
pH umbilical artery	10	210	7.26	0.10	0.01	7.24	7.27	6.84	7.44		.01
	50	60	7.29	0.07	0.01	7.27	7.31	7.07	7.46		
	90	28	7.28	0.08	0.01	7.25	7.31	7.10	7.44		
	Total	298	7.27	0.09	0.01	7.25	7.28	6.84	7.46	.007	
Gestational age from last menstrual period	10	213	35.03	2.38	0.16	34.71	35.35	27.00	37.00		
	50	61	34.03	3.16	0.40	33.22	34.84	25.00	37.00		
	90	28	34.54	3.06	0.58	33.35	35.72	28.00	37.00		
	Total	302	34.78	2.64	0.15	34.48	35.08	25.00	37.00	.072	
Weight (g)	10	213	1797	530	36	1725	1868	350	2560		.001
	50	61	2409	712	91	2226	2591	730	3220		
	90	28	3241	840	159	2915	3566	1450	4800		
	Total	302	2054	752	43	1969	2139	350	4800	.001	
Length (cm)	10	213	43.02	4.68	0.32	42.38	43.65	25.00	51.00		.001
	50	61	46.11	4.86	0.62	44.86	47.35	31.00	52.00		
	90	28	49.80	3.99	0.75	48.26	51.35	40.50	57.00		
	Total	302	44.27	5.12	0.29	43.69	44.85	25.00	57.00	.001	
Head circumference (cm)	10	212	30.40	2.91	0.20	30.01	30.79	21.00	39.00		.001
	50	61	31.89	3.16	0.40	31.08	32.70	22.00	37.00		
	90	28	33.56	3.33	0.63	32.27	34.85	22.00	38.00		
	Total	301	31.00	3.16	0.18	30.64	31.35	21.00	39.00	.001	
*p*TPMDS	10	209	−1.39	0.84	0.06	−1.51	−1.28	−3.99	0.04		.001
	50	60	−0.47	0.70	0.09	−0.66	−0.29	−2.12	0.58		
	90	28	0.28	0.84	0.16	−0.05	0.60	−1.98	2.12		
	Total	297	−1.05	0.99	0.06	−1.16	−0.94	−3.99	2.12	.001	
*p*Intelligence quotient (z*p*IQ)	10	209	−1.79	1.42	0.10	−1.98	−1.60	−6.70	0.95		.001
	50	60	−0.44	0.98	0.13	−0.70	−0.19	−3.58	1.10		
	90	28	0.36	1.33	0.25	−0.16	0.87	−4.54	2.76		
	Total	297	−1.32	1.53	0.09	−1.49	−1.14	−6.70	2.76	.001	
*p*Porteus Maze test (z*p*PMT)	10	210	−2.07	0.79	0.05	−2.18	−1.96	−4.11	−0.99		.001
	50	60	−1.18	1.01	0.13	−1.44	−0.92	−3.46	−0.01		
	90	28	−0.01	1.24	0.23	−0.49	0.46	−2.68	2.27		
	Total	298	−1.70	1.10	0.06	−1.82	−1.57	−4.11	2.27	.001	
*p*Neurologic examination optimality score (*p*zNOS)	10	210	−0.33	0.68	0.05	−0.42	−0.23	−2.30	1.39		.001
	50	60	0.20	0.57	0.07	0.06	0.35	−1.49	1.41		
	90	28	0.49	0.54	0.10	0.28	0.70	−0.72	1.33		
	Total	298	−0.14	0.71	0.04	−0.22	−0.06	−2.30	1.41	.001	
Morphometric vitality index (*cMVI)*	10	209	−1.82	1.05	0.07	−1.96	−1.68	−4.82	−0.08		.001
	50	59	−1.10	1.17	0.15	−1.40	−0.79	−4.38	0.04		
	90	27	−0.17	1.10	0.21	−0.60	0.27	−3.04	1.04		
	Total	295	−1.52	1.19	0.07	−1.66	−1.39	−4.82	1.04	.001	
*p*Developmental disability index *(*pDDI)	10	207	1.19	0.77	0.05	1.09	1.30	−0.53	3.66		.001
	50	58	0.61	0.63	0.08	0.44	0.77	−0.57	2.49		
	90	27	0.22	0.66	0.13	−0.05	0.48	−1.19	1.84		
	Total	292	0.99	0.81	0.05	0.89	1.08	−1.19	3.66	.001	
Birthweight percentile	10	213	3.75	3.02	0.21	3.34	4.16	0.00	9.97		.001
	50	61	54.35	2.85	0.36	53.62	55.08	50.06	59.87		
	90	28	95.10	3.23	0.61	93.85	96.35	90.18	100.00		
	Total	302	22.44	30.88	1.78	18.94	25.94	0.00	100.00	.001	
Birth length percentile	10	213	11.84	12.91	0.88	10.09	13.58	0.00	72.60		.001
	50	61	53.06	18.45	2.36	48.34	57.79	3.25	83.24		
	90	28	82.35	16.72	3.16	75.86	88.83	41.02	100.00		
	Total	302	26.70	28.20	1.62	23.51	29.90	0.00	100.00	.001	
Head circumference percentile	10	212	15.26	19.44	1.34	12.63	17.89	0.00	99.98		.001
	50	61	52.84	21.62	2.77	47.30	58.38	17.00	99.79		
	90	28	73.12	30.56	5.78	61.27	84.97	1.64	100.00		
	Total	301	28.26	29.55	1.70	24.90	31.61	0.00	100.00	.001	

*PIVH*, Peri-/intraventricular hemorrhage; *pTPMDS, predicted* total psychomotor development score; *WMD*, White matter damage

**Table 2:** Obstetrical risk factors in preterm infants (24–37 weeks’ gestation; n=302) derived from a prospective cranial ultrasound screening database (n=5,301) in 3 birthweight percentile groups (ie, small [10%], appropriate [50%], and large [90%] for gestational age)

						95% confidence interval				Welch test	Games–Howell
		N		Standard	Standard						
Obstetrical risk factors	Percentile (%)		Mean	deviation	error	Lower limit	Upper limit	Minimum	Maximum	*P* value	*P* value
Maternal age (y)	10	213	27.33	5.23	0.36	26.62	28.04	16.00	41.00		
	50	61	27.75	5.51	0.71	26.34	29.16	17.00	42.00		
	90	28	28.43	5.08	0.96	26.46	30.40	21.00	39.00		
	Total	302	27.52	5.27	0.30	26.92	28.11	16.00	42.00	.531	
Parity	10	213	1.52	0.75	0.05	1.42	1.62	1.00	5.00		
	50	61	1.66	0.83	0.11	1.44	1.87	1.00	5.00		
	90	28	2.29	1.78	0.34	1.59	2.98	1.00	9.00		
	Total	302	1.62	0.93	0.05	1.51	1.72	1.00	9.00	.059	
EPH syndrome	10	213	0.25	0.43	0.03	0.19	0.31	0.00	1.00		.007
	50	61	0.10	0.30	0.04	0.02	0.18	0.00	1.00		
	90	28	0.07	0.26	0.05	−0.03	0.17	0.00	1.00		
	Total	302	0.20	0.40	0.02	0.16	0.25	0.00	1.00	.001	
PROM	10	213	0.21	0.41	0.03	0.15	0.26	0.00	1.00		.001
	50	61	0.36	0.48	0.06	0.24	0.48	0.00	1.00		
	90	28	0.61	0.50	0.09	0.41	0.80	0.00	1.00		
	Total	302	0.27	0.45	0.03	0.22	0.33	0.00	1.00	.001	
Diabetes mellitus (gestational)	10	213	0.01	0.12	0.01	0.00	0.03	0.00	1.00		
	50	61	0.05	0.22	0.03	−0.01	0.11	0.00	1.00		
	90	28	0.18	0.39	0.07	0.03	0.33	0.00	1.00		
	Total	302	0.04	0.19	0.01	0.02	0.06	0.00	1.00	.055	
Vaginal bleeding	10	213	0.15	0.50	0.03	0.09	0.22	0.00	2.00		
	50	61	0.18	0.50	0.06	0.05	0.31	0.00	2.00		
	90	28	0.11	0.42	0.08	−0.05	0.27	0.00	2.00		
	Total	302	0.16	0.49	0.03	0.10	0.21	0.00	2.00	.772	
Amnion infection	10	213	0.00	0.00	0.00	0.00	0.00	0.00	0.00		
	50	61	0.00	0.00	0.00	0.00	0.00	0.00	0.00		
	90	28	0.00	0.00	0.00	0.00	0.00	0.00	0.00		
	Total	302	0.00	0.00	0.00	0.00	0.00	0.00	0.00		
Hypertension	10	212	0.91	0.29	0.02	0.87	0.95	0.00	1.00		.001
	50	60	0.98	0.13	0.02	0.95	1.02	0.00	1.00		
	90	28	1.00	0.00	0.00	1.00	1.00	1.00	1.00		
	Total	300	0.93	0.25	0.01	0.90	0.96	0.00	1.00		
Hypotension	10	194	0.99	0.07	0.01	0.98	1.01	0.00	1.00		
	50	60	0.98	0.13	0.02	0.95	1.02	0.00	1.00		
	90	28	1.00	0.00	0.00	1.00	1.00	1.00	1.00		
	Total	282	0.99	0.08	0.01	0.98	1.00	0.00	1.00		
Multiple pregnancy	10	213	0.26	0.44	0.03	0.20	0.32	0.00	1.00		.003
	50	61	0.10	0.30	0.04	0.02	0.18	0.00	1.00		
	90	28	0.07	0.26	0.05	−0.03	0.17	0.00	1.00		
	Total	302	0.21	0.41	0.02	0.17	0.26	0.00	1.00	.001	
IUGR	10	213	0.31	0.47	0.03	0.25	0.38	0.00	1.00		.001
	50	61	0.00	0.00	0.00	0.00	0.00	0.00	0.00		
	90	28	0.00	0.00	0.00	0.00	0.00	0.00	0.00		
	Total	302	0.22	0.42	0.02	0.17	0.27	0.00	1.00		
Pathologic cardiotocography	10	213	0.43	0.50	0.03	0.36	0.49	0.00	1.00		.003
	50	61	0.21	0.41	0.05	0.11	0.32	0.00	1.00		
	90	28	0.21	0.42	0.08	0.05	0.38	0.00	1.00		
	Total	302	0.36	0.48	0.03	0.31	0.42	0.00	1.00	.001	
Meconium-stained amniotic fluid	10	213	0.02	0.15	0.01	0.00	0.04	0.00	1.00		
	50	61	0.00	0.00	0.00	0.00	0.00	0.00	0.00		
	90	28	0.04	0.19	0.04	−0.04	0.11	0.00	1.00		
	Total	302	0.02	0.14	0.01	0.00	0.04	0.00	1.00		
Prolonged or arrested labor	10	213	0.07	0.26	0.02	0.04	0.11	0.00	1.00		
	50	61	0.10	0.30	0.04	0.02	0.18	0.00	1.00		
	90	28	0.18	0.39	0.07	0.03	0.33	0.00	1.00		
	Total	302	0.09	0.28	0.02	0.05	0.12	0.00	1.00	.326	
Mode of delivery	10	213	1.82	0.78	0.05	1.71	1.92	1.00	5.00		
	50	61	1.92	1.10	0.14	1.64	2.20	1.00	5.00		
	90	28	1.71	0.76	0.14	1.42	2.01	1.00	4.00		
	Total	302	1.83	0.85	0.05	1.73	1.92	1.00	5.00	.606	
Presentation	10	213	1.49	0.86	0.06	1.38	1.61	1.00	3.00		
	50	61	1.43	0.81	0.10	1.22	1.63	1.00	3.00		
	90	28	1.29	0.71	0.13	1.01	1.56	1.00	3.00		
	Total	302	1.46	0.83	0.05	1.37	1.55	1.00	3.00	.364	
pCO2 umbilical artery	10	209	46.69	11.47	0.79	45.12	48.25	22.40	114.40		.009
	50	60	42.31	9.52	1.23	39.85	44.77	26.70	76.10		
	90	28	44.95	6.33	1.20	42.50	47.41	30.20	56.00		
	Total	297	45.64	10.83	0.63	44.40	46.88	22.40	114.40	.014	
Base deficit umbilical artery	10	207	7.77	4.83	0.34	7.10	8.43	0.30	25.20		
	50	59	6.97	4.02	0.52	5.92	8.01	0.10	15.00		
	90	28	7.21	4.40	0.83	5.51	8.92	0.20	18.20		
	Total	294	7.55	4.63	0.27	7.02	8.08	0.10	25.20	.418	
pO2 umbilical artery	10	207	15.15	5.65	0.39	14.37	15.92	3.60	35.60		.001
	50	59	18.36	5.18	0.67	17.01	19.71	4.50	31.80		
	90	28	17.89	4.83	0.91	16.01	19.76	6.40	27.10		
	Total	294	16.05	5.65	0.33	15.40	16.70	3.60	35.60	.001	
Transfer to NICU	10	110	0.20	0.40	0.04	0.12	0.28	0.00	1.00		
	50	36	0.14	0.35	0.06	0.02	0.26	0.00	1.00		
	90	10	0.20	0.42	0.13	−0.10	0.50	0.00	1.00		
	Total	156	0.19	0.39	0.03	0.12	0.25	0.00	1.00	.688	

*EPH*, edema, proteinuria, and hypertension; *IUGR*, intrauterine growth restriction; *NICU*, neonatal intensive care unit; *PROM*, premature rupture of membranes.

**Figure 1:**
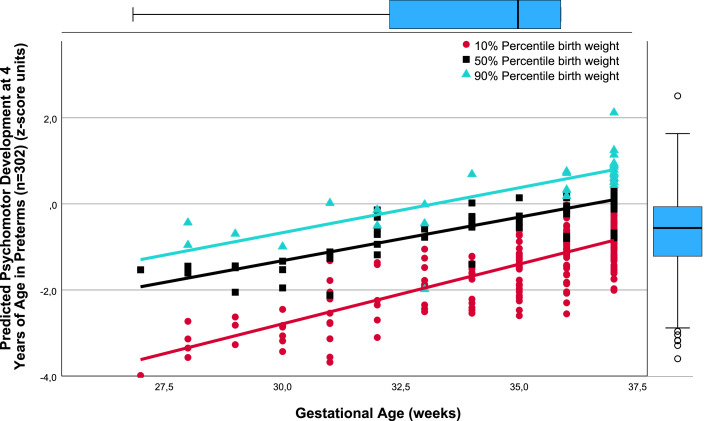
The correlations between predicted pTPMDS (z-score) units and Gestational age (weeks) in three groups of birth weight percentiles SGA, AGA, and LGA (10%, 50%, 90%) The correlations between *predicted* (*p*) TPMDS (z-score) units and gestational age (weeks) in 3 groups of birthweight percentiles (small [10%], appropriate [50%], and large [90%] for gestational age) in 302 preterm newborns born at ≤37^+6/7^ weeks of gestation are depicted (z*p*TPMDS_10=−11.102+0.277 × gestational week; *r*=0.785; SE estimate=0.523; n=209; *P*<.001; z*p*TPMDS_50=−7.385+0.202 × gestational week; *r*=0.850; SE estimate=0.374; n=60; *P*<.001; z*p*TPMDS_90=−6.930+0.209 × gestational week; *r*=0.765; SE estimate=0.548; n=28; *P*<.001). *p*TPMDS represents the average of *predicted* IQ, PMT score, and NOS determined at 4.3 (SD, 0.8) years of age (z*p*TPMDS=(z*p*IQ+z*p*PMT result+z*p*NOS)/3).^1^^,^^3^^,^^22^ Of note, the slope (*P*<.01) and intercept of the regression line of growth-restricted preterm newborns (10% percentile group) are significantly different from those of immature newborns without growth restriction (50%) (*P*<.01). This is clinically relevant because growth restriction at lower gestational age is more harmful to the brain, necessitating prospective obstetrical risk management (eg, by detecting brain-sparing in Doppler flow analysis of the middle cerebral artery) and early intervention to ameliorate adverse effects on neurocognition in childhood.^21^ *IQ*, intelligence quotient; *NOS*, neurologic examination optimality score; *PMT*, Porteus Maze test; *SD*, standard deviation; *SE*, standard error; *TPMDS*, total psychomotor development score.

**Figure 2:**
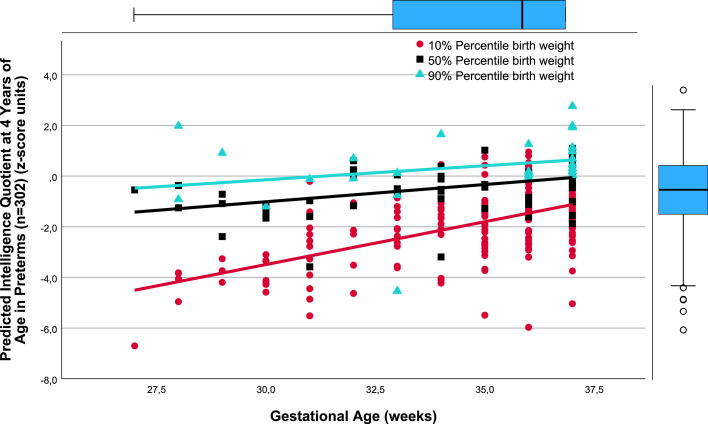
The correlations between predicted Intelligence quotient at 4.3 years (SD, 0.8) of age (z- score units) and Gestational age (weeks) in three groups of birth weight percentiles SGA, AGA, and LGA (10%, 50%, 90%) The correlations between *predicted* IQ at 4.3 (SD, 0.8) years of age (z-score units) and gestational age (weeks) in 3 groups of birthweight percentiles (small [10%], appropriate [50%], and large [90%] for gestational age) in 302 preterm newborns born at ≤37^+6/7^ weeks of gestation are depicted (z*p*IQ_10=−13.633+0.338 × gestational week; *r*=0.569, SE estimate=1.167; n=209; *P*<.001; z*p*IQ_50=−5.098+0.136 × gestational week; *r*=0.413; SE estimate=0.896; n=60; *P*<.001; z*p*IQ_90=−3.481+0.111 × gestational week; *r*=0.256; SE estimate=1.311; n=28; not significant).^1^^,^^3^^,^^22^ The slope (*P*<.001) and intercept of the regression line of growth-restricted preterm newborns (10% percentile group) are significantly different from those of immature newborns without growth restriction (50%) (*P*<.001). This is clinically important because growth restriction at lower gestational age is more harmful to the brain, necessitating prospective obstetrical risk management (eg, by detecting brain-sparing in Doppler flow analysis of the middle cerebral artery) and early intervention to ameliorate adverse effects on cognition.^21^ *IQ*, intelligence quotient; *SE*, standard error.

**Figure 3:**
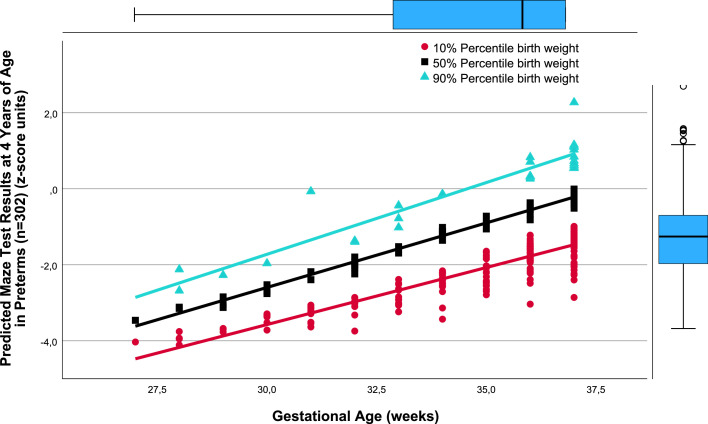
The correlations between predicted Maze test results (z-score units) at 4.3 years (SD, 0.8) of age and Gestational age (weeks) in three groups of birth weight percentiles SGA, AGA, and LGA The correlations between *predicted* PMT results (z-score units) at 4.3 (SD, 0.8) years of age and gestational age (weeks) in 3 groups of birthweight percentiles (small [10%], appropriate [50%], and large [90%] for gestational age) in 302 preterm newborns born at ≤37^+6/7^ weeks of gestation are depicted (z*p*PMT_10=−12.580+0.300 × gestational week; *r*=0.904; SE estimate=0.341; n=210; *P*<.001; z*p*PMT_50=−12.788+0.340 × gestational week; *r*=0.991; SE estimate=0.138; n=60; *P*<.001; z*p*PMT_90=−13.052+0.378 × gestational week; *r*=0.935; SE estimate=0.448; n=28; *P*<.001).^1^^,^^3^^,^^22^ The slope (*P*<.003) and intercept of the regression line of growth-restricted preterm newborns (10% percentile group) are significantly different from those of immature newborns without growth restriction (50%) (*P*<.007). The slope (*P*<.023) and intercept of the regression line of the large for gestational age preterm infants (90%) are significantly different from those of immature newborns without growth restriction (50%) (*P*<.024). Reduced *predicted* PMT results in growth-restricted preterm infants are clinically relevant because PMT domains are considered largely independent of standard intelligence quotient testing because of its untimed, configural, and problem-solving task. Furthermore, the PMT is a uniquely sensitive measure of executive function ability, comprising the domains of fine motor ability, dexterity, planning capacity, stability, and learning ability.^1^^,^^3^^,^^22^ *PMT*, Porteus Maze test; *SD*, standard deviation; *SE*, standard error.

**Figure 4:**
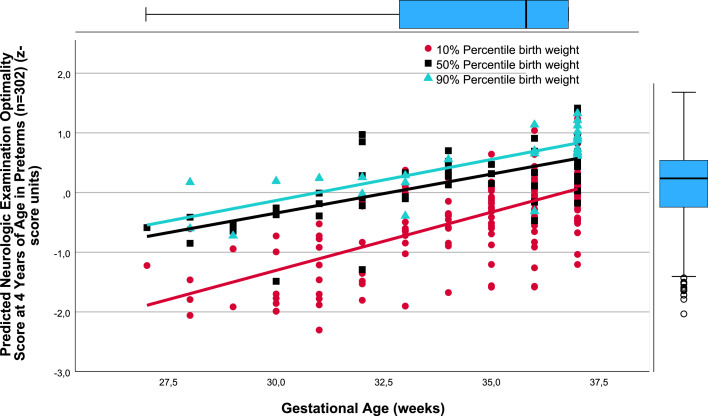
The correlations between predicted Neurologic examination optimality score at 4.3 years (SD, 0.8) of age (z-score units) and Gestational age (weeks) in three groups of birth weight percentiles SGA, AGA, and LGA The correlations between *predicted* NOS at 4.3 (SD, 0.8) years of age (z-score units) and gestational age (weeks) in 3 groups of birthweight percentiles (small [10%], appropriate [50%], and large [90%] for gestational age) in 302 preterm newborns born at ≤37^+6/7^ weeks of gestation are depicted (z*p*NOS_10=−7.156+0.195 × gestational week; *r*=0.686; SE estimate=0.496; n=210; *P*<.001; z*p*NOS_50=−4.268+0.131 × gestational week; *r*=0.681; SE estimate=0.420; n=60; *P*<.001; z*p*NOS_90=−4.257+0.137 × gestational week; *r*=0.776; SE estimate=0.438; n=28; *P*<.001).^1^^,^^3^^,^^22^ The slope (*P*<.003) and intercept of the regression line of growth-restricted preterm newborns (10% percentile group) are significantly different from those of immature newborns without growth restriction (50%) (*P*<.0001). This is clinically important because growth restriction at lower gestational age particularly affects motor performance in childhood, necessitating early intervention by neurorehabilitation.^1^^,^^3^^,^^22^ *NOS*, neurologic examination optimality score; *SE*, standard error.

**Figure 5:**
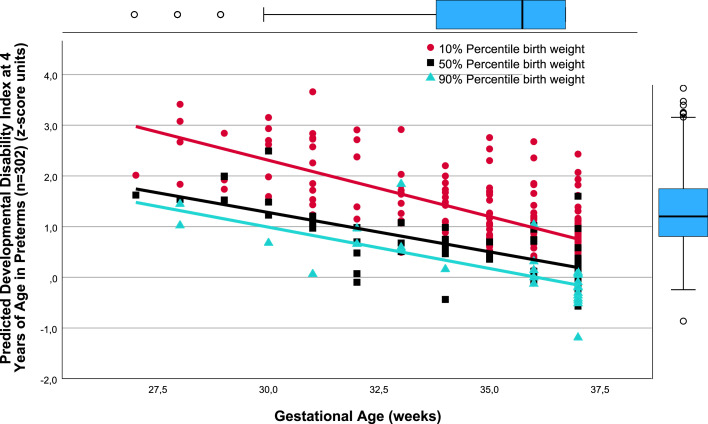
The correlations between predicted Developmental disability index determined at 4.3 years (SD, 0.8) (z-score units) and Gestational age (weeks) in three groups of birth weight percentiles SGA, AGA, and LGA The correlations between *predicted* DDI determined at 4.3 (SD, 0.8) years of age (z-score units) and gestational age (weeks) in 3 groups of birthweight percentiles (small [10%], appropriate [50%], and large [90%] for gestational age) in 302 preterm newborns born at ≤37^+6/7^ weeks of gestation are depicted (*p*DDI_10=8.969−0.222 × gestational week; *r*=0.690; SE estimate=0.559; n=207; *P*<.001; *p*DDI_50=5.932−0.155 × gestational week; *r*=0.707; SE estimate=0.451; n=58; *P*<.001; *p*DDI_90=5.882−0.163 × gestational week; *r*=0.719; SE estimate=0.469; n=27; *P*<.001).^1^^,^^3^^,^^22^ The slope (*P*<.009) and intercept of the regression line of growth-restricted preterm newborns (10% percentile group) are significantly different (*P*<.0008) from those of immature newborns without growth restriction (50%). These results underscore the significance of detecting growth restriction in preterm infants for predicting developmental trajectories and the degree of *predicted* disability at 4 years of age, whereby the probability of future disability increases with decreasing gestational age.^1^^,^^3^^,^^22^ *DDI*, developmental disability index; *SD*, standard deviation; *SE*, standard error.

There was a close relation between gestational age (weeks) and *predicted* TPMDS in each of the SGA, AGA, and LGA birthweight percentile groups (10%, 50%, and 90%). Namely, lower gestational age was associated with poorer yields in the composite TPMDS (Figure 1), *predicted* IQ (Figure 2), *predicted* PMT results (Figure 3), and *predicted* NOS (Figure 4). Furthermore, the *predicted* DDI was negatively correlated with gestational weeks (Figure 5). These results underscore the significance of birthweight percentiles for identification of differential effects of both growth restriction and immaturity in predicting developmental trajectories at 4 years of age (Table 3).

Jensen. Newborn growth restriction and psychomotor development. Am J Obstet Gynecol Glob Rep 2024.

Jensen. Newborn growth restriction and psychomotor development. Am J Obstet Gynecol Glob Rep 2024.

Jensen. Newborn growth restriction and psychomotor development. Am J Obstet Gynecol Glob Rep 2024.

Jensen. Newborn growth restriction and psychomotor development. Am J Obstet Gynecol Glob Rep 2024.

Jensen. Newborn growth restriction and psychomotor development. Am J Obstet Gynecol Glob Rep 2024.

**Table 3:** Relation between psychomotor development indices and measures and gestational week at birth defined by date of last menstrual period in 3 birthweight percentile groups (ie, small [10%], appropriate [50%], and large [90%] for gestational age)

Percentile	Variable		Relation to gestational wk	r	SE estimate	n	*P* value
birthweight (%)							
10	z*p*TPMDS_10	=	−11.102+0.277 × gestational wk	0.785	0.523	209	<.001
50	z*p*TPMDS_50	=	−7.385+0.202 × gestational wk	0.850	0.374	60	<.001
90	z*p*TPMDS_90	=	−6.930+0.209 × gestational wk	0.765	0.548	28	<.001
10	z*p*IQ_10	=	−13.633+0.338 × gestational wk	0.569	1.167	209	<.001
50	z*p*IQ_50	=	−5.098+0.136 × gestational wk	0.413	0.896	60	<.001
90	z*p*IQ_90	=	−3.481+0.111 × gestational wk	0.256	1.311	28	n.s.
10	z*p*PMT_10	=	−12.580+0.300 × gestational wk	0.904	0.341	210	<.001
50	z*p*PMT_50	=	−12.788+0.340 × gestational wk	0.991	0.138	60	<.001
90	z*p*PMT_90	=	−13.052+0.378 × gestational wk	0.935	0.448	28	<.001
10	z*p*NOS_10	=	−7.156+0.195 × gestational wk	0.686	0.496	210	<.001
50	z*p*NOS_50	=	−4.268+0.131 × gestational wk	0.681	0.420	60	<.001
90	z*p*NOS_90	=	−4.257+0.137 × gestational wk	0.776	0.438	28	<.001
10	*p*DDI_10	=	8.969−0.222 × gestational wk	0.690	0.559	207	<.001
50	*p*DDI_50	=	5.932−0.155 × gestational wk	0.707	0.451	58	<.001
90	*p*DDI_90	=	5.882−0.163 × gestational wk	0.719	0.469	27	<.001

*DDI*, developmental disability index; *IQ*, intelligence quotient; *NOS*, neurologic examination optimality score; *n.s.*, nonsignificant; *p*, predicted; *PMT*, Porteus Maze test; *TPMDS*, total psychomotor development score; *z*, z-score.

Using birthweight percentiles as continuous variables combined with gestational age in all 854 preterm infants allows for full range prediction of psychomotor indices and measures (Table 4). For example, TPMDS of infants born preterm can be predicted using gestational age and *c*MVI as independent variables (zpTPMDS=1.372−0.036 × gestational week+0.689 × *c*MVI; *r*=0.812; SE estimate=0.518; n=829; *P*<.001).

**Table 4:** Relation between psychomotor development indices and measures and gestational week at birth defined by date of last menstrual period and birthweight percentile allowing for full range prediction (n=854)

Variable		Relation to gestational wk and birthweight percentile	r	SE estimate	n	*P* value
						
z*p*TPMDS	=	−8.916+0.218 × gestational wk+0.018 × birthweight percentile	0.849	0.469	837	<.001
z*p*IQ	=	−8.051+0.186 × gestational wk+0.024 × birthweight percentile	0.585	1.092	838	<.001
z*p*PMT	=	−13.297+0.322 × gestational wk+0.021 × birthweight percentile	0.974	0.243	841	<.001
z*p*NOS	=	−5.427+0.148 × gestational wk+0.009 × birthweight percentile	0.715	0.461	839	<.001
z*p*DDI	=	7.405−0.179 × gestational wk−0.011 × birthweight percentile	0.733	0.520	832	<.001

*DDI*, developmental disability index; *IQ*, intelligence quotient; *NOS*, neurologic examination optimality score; *p*, predicted; *PMT*, Porteus Maze test; *TPMDS*, total psychomotor development score; *z*, z-score.

The differential effects on psychomotor development in SGA newborns with IUGR (n=67) and those without (n=146) are shown in Table 5. Gestational age, acid–base balance, blood gases, and Apgar scores were not different between the groups. However, in the group with IUGR, there were significantly poorer results in psychomotor development indices (z*p*TPMDS, MVI, *p*DDI) and measures (z*p*IQ, *p*zNOS) and higher rates of peri- and intraventricular hemorrhage (*P*<.05) and white matter damage (*P*<.05), reduced morphometric measures, and lower percentiles for birthweight, length, and head circumference, clearly indicating IUGR as the most important threat to *predicted* psychomotor development.

**Table 5:** Obstetrical and birth-related risk factors and psychomotor development indices and measures in small for gestational age preterm infants (birthweight percentile group 10%; 24–37 weeks’ gestation; n=213) without (n=146) and with (n=67) intrauterine growth restriction derived from a prospective cranial ultrasound screening database (n=5,301)

Variable	Group	N	Mean	Standard deviation	95% confidence interval of the mean		Minimum	Maximum	Welch test
					Lower limit	Upper limit			*P* value
Maternal age (y)	SGA	146	27.77	5.14	26.93	28.61	16.00	41.00	
	IUGR	67	26.37	5.35	25.07	27.68	16.00	40.00	ns
	Total	213	27.33	5.23	26.62	28.04	16.00	41.00	
Parity	SGA	146	1.63	0.83	1.49	1.77	1.00	5.00	
	IUGR	67	1.28	0.45	1.17	1.39	1.00	2.00	<.001
	Total	213	1.52	0.75	1.42	1.62	1.00	5.00	
Gestational age (wk from last menstrual period)	SGA	146	35.12	2.27	34.75	35.50	28.00	37.00	
	IUGR	67	34.82	2.59	34.19	35.45	27.00	37.00	ns
	Total	213	35.03	2.38	34.71	35.35	27.00	37.00	
EPH syndrome	SGA	146	0.21	0.41	0.15	0.28	0.00	1.00	
	IUGR	67	0.33	0.47	0.21	0.44	0.00	1.00	ns
	Total	213	0.25	0.43	0.19	0.31	0.00	1.00	
PROM	SGA	146	0.23	0.42	0.16	0.29	0.00	1.00	
	IUGR	67	0.16	0.37	0.07	0.26	0.00	1.00	ns
	Total	213	0.21	0.41	0.15	0.26	0.00	1.00	
Diabetes mellitus (gestational)	SGA	146	0.01	0.12	−0.01	0.03	0.00	1.00	
	IUGR	67	0.01	0.12	−0.01	0.04	0.00	1.00	ns
	Total	213	0.01	0.12	0.00	0.03	0.00	1.00	
Vaginal bleeding	SGA	146	0.16	0.53	0.08	0.25	0.00	2.00	
	IUGR	67	0.13	0.46	0.02	0.25	0.00	2.00	ns
	Total	213	0.15	0.50	0.09	0.22	0.00	2.00	
Amnion infection	SGA	146	0.00	0.00	0.00	0.00	0.00	0.00	
	IUGR	67	0.00	0.00	0.00	0.00	0.00	0.00	ns
	Total	213	0.00	0.00	0.00	0.00	0.00	0.00	
Hypotension	SGA	145	0.94	0.23	0.91	0.98	0.00	1.00	
	IUGR	67	0.84	0.37	0.74	0.93	0.00	1.00	.030
	Total	212	0.91	0.29	0.87	0.95	0.00	1.00	
Hypertension	SGA	138	0.99	0.09	0.98	1.01	0.00	1.00	
	IUGR	56	1.00	0.00	1.00	1.00	1.00	1.00	ns
	Total	194	0.99	0.07	0.98	1.01	0.00	1.00	
Multiples	SGA	146	0.36	0.48	0.28	0.43	0.00	1.00	
	IUGR	67	0.06	0.24	0.00	0.12	0.00	1.00	<.001
	Total	213	0.26	0.44	0.20	0.32	0.00	1.00	
Pathologic cardiotocography	SGA	146	0.36	0.48	0.28	0.43	0.00	1.00	
	IUGR	67	0.58	0.50	0.46	0.70	0.00	1.00	.002
	Total	213	0.43	0.50	0.36	0.49	0.00	1.00	
Meconium-stained amniotic fluid	SGA	146	0.03	0.16	0.00	0.05	0.00	1.00	
	IUGR	67	0.01	0.12	−0.01	0.04	0.00	1.00	ns
	Total	213	0.02	0.15	0.00	0.04	0.00	1.00	
Prolonged or arrested labor	SGA	146	0.08	0.26	0.03	0.12	0.00	1.00	
	IUGR	67	0.06	0.24	0.00	0.12	0.00	1.00	ns
	Total	213	0.07	0.26	0.04	0.11	0.00	1.00	
Mode of delivery	SGA	146	1.78	0.83	1.64	1.92	1.00	5.00	
	IUGR	67	1.90	0.63	1.74	2.05	1.00	4.00	ns
	Total	213	1.82	0.78	1.71	1.92	1.00	5.00	
Presentation	SGA	146	1.50	0.86	1.36	1.64	1.00	3.00	
	IUGR	67	1.48	0.86	1.27	1.69	1.00	3.00	ns
	Total	213	1.49	0.86	1.38	1.61	1.00	3.00	
Sex	SGA	146	1.49	0.50	1.41	1.58	1.00	2.00	
	IUGR	67	1.57	0.50	1.45	1.69	1.00	2.00	ns
	Total	213	1.52	0.50	1.45	1.58	1.00	2.00	
Weight (g)	SGA	146	1884	513	1800	1968	480	2560	
	IUGR	67	1605	519	1479	1732	350	2370	<.001
	Total	213	1797	530	1725	1868	350	2560	
Length (cm)	SGA	146	43.65	4.43	42.92	44.37	26.00	51.00	
	IUGR	67	41.64	4.93	40.44	42.84	25.00	50.00	.005
	Total	213	43.02	4.68	42.38	43.65	25.00	51.00	
Head circumference (cm)	SGA	145	30.77	2.76	30.31	31.22	22.00	39.00	
	IUGR	67	29.61	3.07	28.86	30.36	21.00	37.00	.009
	Total	212	30.40	2.91	30.01	30.79	21.00	39.00	
Apgar 1 min	SGA	145	7.17	2.03	6.84	7.51	1.00	10.00	
	IUGR	67	6.63	2.26	6.08	7.18	1.00	9.00	ns
	Total	212	7.00	2.11	6.71	7.29	1.00	10.00	
Apgar 5 min	SGA	146	8.80	1.36	8.58	9.02	3.00	10.00	
	IUGR	66	8.42	1.36	8.09	8.76	4.00	10.00	ns
	Total	212	8.68	1.37	8.50	8.87	3.00	10.00	
Apgar 10 min	SGA	143	9.36	0.92	9.21	9.52	6.00	10.00	
	IUGR	67	9.13	0.95	8.90	9.37	7.00	10.00	ns
	Total	210	9.29	0.93	9.16	9.42	6.00	10.00	
pH umbilical artery	SGA	145	7.26	0.10	7.24	7.27	6.84	7.43	
	IUGR	65	7.25	0.09	7.23	7.27	7.02	7.44	ns
	Total	210	7.26	0.10	7.24	7.27	6.84	7.44	
pCO2 umbilical artery	SGA	145	46.36	11.59	44.46	48.26	22.40	114.40	
	IUGR	64	47.43	11.27	44.62	50.24	26.30	76.10	ns
	Total	209	46.69	11.47	45.12	48.25	22.40	114.40	
Base deficit umbilical artery	SGA	144	7.50	4.95	6.68	8.31	0.30	25.20	
	IUGR	63	8.38	4.51	7.25	9.52	0.30	22.40	ns
	Total	207	7.77	4.83	7.10	8.43	0.30	25.20	
pO2 umbilical artery	SGA	144	15.32	5.86	14.36	16.29	3.60	35.60	
	IUGR	63	14.75	5.18	13.45	16.06	5.40	29.00	ns
	Total	207	15.15	5.65	14.37	15.92	3.60	35.60	
PIVH present	SGA	146	0.16	0.37	0.10	0.22	0.00	1.00	
	IUGR	67	0.28	0.45	0.17	0.39	0.00	1.00	.049
	Total	213	0.20	0.40	0.14	0.25	0.00	1.00	
Transfer to NICU	SGA	72	0.14	0.35	0.06	0.22	0.00	1.00	
	IUGR	38	0.32	0.47	0.16	0.47	0.00	1.00	.046
	Total	110	0.20	0.40	0.12	0.28	0.00	1.00	
WMD present	SGA	146	0.13	0.34	0.07	0.19	0.00	1.00	
	IUGR	67	0.27	0.45	0.16	0.38	0.00	1.00	.026
	Total	213	0.17	0.38	0.12	0.23	0.00	1.00	
pIntelligence quotient (zpIQ)	SGA	144	−1.62	1.30	−1.83	−1.40	−5.04	0.95	
	IUGR	65	−2.17	1.58	−2.57	−1.78	−6.70	0.80	.015
	Total	209	−1.79	1.42	−1.98	−1.60	−6.70	0.95	
pPorteus Maze test (zpPMT)	SGA	145	−2.01	0.79	−2.14	−1.88	−4.11	−0.99	
	IUGR	65	−2.21	0.80	−2.41	−2.01	−4.03	−0.99	.090
	Total	210	−2.07	0.79	−2.18	−1.96	−4.11	−0.99	
pNeurologic examination optimality score (pzNOS)	SGA	145	−0.22	0.64	−0.33	−0.12	−2.30	1.39	
	IUGR	65	−0.56	0.71	−0.73	−0.38	−1.99	0.64	<.001
	Total	210	−0.33	0.68	−0.42	−0.23	−2.30	1.39	
pTotal psychomotor development (pzTPMDS)	SGA	144	−1.28	0.79	−1.41	−1.15	−3.57	0.04	
	IUGR	65	−1.65	0.90	−1.87	−1.42	−3.99	−0.20	.005
	Total	209	−1.39	0.84	−1.51	−1.28	−3.99	0.04	
cMorphometric vitality index (cMVI)	SGA	142	−1.66	1.00	−1.82	−1.49	−4.82	−0.08	
	IUGR	67	−2.16	1.10	−2.42	−1.89	−4.72	−0.66	.002
	Total	209	−1.82	1.05	−1.96	−1.68	−4.82	−0.08	
pDevelopmental disability index (pDDI)	SGA	142	1.09	0.73	0.97	1.22	−0.53	3.66	
	IUGR	65	1.41	0.82	1.21	1.61	0.14	3.15	.009
	Total	207	1.19	0.77	1.09	1.30	−0.53	3.66	
Weight/head circumference ratio	SGA	145	60.42	12.98	58.29	62.55	21.82	77.78	
	IUGR	67	53.07	13.84	49.70	56.45	16.67	74.19	<.001
	Total	212	58.10	13.66	56.25	59.95	16.67	77.78	
Birthweight percentile (%)	SGA	146	4.68	2.99	4.19	5.17	0.00	9.97	
	IUGR	67	1.73	1.92	1.26	2.20	0.00	8.15	<.001
	Total	213	3.75	3.02	3.34	4.16	0.00	9.97	
Birth length percentile (%)	SGA	146	13.86	13.50	11.66	16.07	0.00	72.60	
	IUGR	67	7.42	10.29	4.91	9.93	0.00	46.89	<.001
	Total	213	11.84	12.91	10.09	13.58	0.00	72.60	
Head circumference percentile (%)	SGA	145	17.90	19.44	14.71	21.09	0.00	99.98	
	IUGR	67	9.55	18.32	5.08	14.02	0.00	99.47	.003
	Total	212	15.26	19.44	12.63	17.89	0.00	99.98	

*EPH*, edema, proteinuria, and hypertension; *IUGR*, intrauterine growth restriction; *NICU*, neonatal intensive care unit; *ns*, nonsignificant; *PIVH*, Peri-/intraventricular hemorrhage; *PROM*, premature rupture of membranes; *SGA*, small for gestational age; *WMD*, White matter damage.

There were also significant differences in psychomotor development indices and measures between newborns with early-onset IUGR at ≤32 weeks of gestation (mean gestational age, 29.9 [SD, 1.5] weeks; n=15) and those with late-onset IUGR (mean gestational age, 35.9 [SD, 1.2] weeks; n=59). The former group had reduced morphometrics and increased rates of brain injury. Furthermore, this group had markedly poorer results in overall *predicted* psychomotor development (z*p*TPMDS=−2.8 [SD, 0.6] vs −1.3 [SD, 0.8]; *P*<.001), DDI (*p*DDI=2.5 [SD, 0.6] vs 1.1 [SD, 0.7]; *P*<.001), MVI (*c*MVI=−3.9 [SD, 1.2] vs −1.7 [SD, 0.8]; *P*<.001), IQ (z*p*IQ=−3.5 [SD, 1.4] vs −1.6 [SD, 1.5]; *P*<.001), PMT (z*p*PMT=−3.4 [SD, 0.3] vs −1.8 [SD, 0.7]; *P*<.001), and NOS (*p*zNOS=−1.5 [SD, 0.5] vs −0.3 [SD, 0.6]; *P*<.001). Almost all pregnancies, in both early and late IUGR cases, were terminated by cesarean delivery (mode of delivery=1.9 [SD, 0.3] vs 1.9 [SD, 0.7]; *P*=.448).

## Discussion

### Principal findings

This study demonstrates that in 854 preterm infants born at ≤37^+6/7^ weeks of gestation from a large prospective cohort (n=5,301), growth restriction compared with immaturity had significantly more harmful effects on predicted psychomotor development at preschool age. This is relevant for clinical management of growth restriction and that of threatened preterm birth as well as for parental consultation, in that gestation can only safely be prolonged in the absence of growth restriction to improve maturation and psychomotor development.^4^, ^5^, ^6^, ^7^, ^8^, ^9^, ^10^, ^11^, ^12^, ^13^, ^14^ In previous accounts, we have established that simple birth variables and obstetrical risk factors allow for predicting neurocognitive development at the preschool age of 4 years.^1^, ^2^, ^3^ However, the differential effects of growth restriction compared with those of immaturity could not be deciphered.^3^

As evidenced by the significantly different slopes and constants of the regressions between the AGA (50%) and SGA (10%) birthweight percentile groups, the harmful effects of growth restriction increase with decreasing gestational age (Figures 1, 2, 4, and 5). Although the specific mechanisms are elusive, structural changes in brain development including reduced gray matter volume in various brain regions may be involved,^15^, ^16^, ^17^, ^18^, ^19^^,^^28^ making the poor neurocognitive development in growth-restricted infants comprehensible relative to infants presenting with immaturity only. Importantly, the detrimental and potentially microstructural effects of growth restriction on the fetal brain and hence on preschool neurocognition are specific and apparently unrelated to cerebral hemorrhage and/or white matter damage.^19^, ^20^, ^21^ This has to be accounted for in clinical obstetrics.

### Clinical implications

The prediction of psychomotor development by indices (*p*TPMDS, *p*DDI, *c*MVI) or measures (*p*IQ, *p*PMT, *p*NOS) at birth has previously been advocated to orchestrate early intervention strategies, timely rehabilitation, or even cell therapies to improve educational success and prevent mental illnesses in childhood and adolescence (eg, male attention deficit hyperactivity disorders and female depression and anxiety disorders).^1^, ^2^, ^3^, ^4^, ^5^^,^^10^ The present results indicating that growth-restricted infants fare far worse in development than solely immature infants at the same gestational age shed light on the urgency to manage growth restriction in clinical obstetrics appropriately in a timely manner.^22^ This holds particularly true for IUGR (Table 5). Further, it is obvious that brain-sparing as a compensatory mechanism caused by circulatory and metabolic centralization when oxygen is at short supply will not suffice to maintain brain homeostasis.^21^^,^^29^, ^30^, ^31^ Thus, brain-sparing does not ensure age-appropriate neurocognition, and it is clear from magnetic resonance imaging that growth-restricted infants represent a high-risk subgroup of infants with a complex and distinct set of microstructural brain abnormalities not observed in appropriately grown preterm infants.^21^ Recent imaging advances even demonstrate more complex changes including altered fiber organization and impaired connectivity networks such as the cortico-basal ganglia-thalamo-cortical loop.^21^ Thus, management in clinical obstetrics has to make the decision when to deliver intrauterine growth–restricted fetuses to ensure oxygen and nutrient supply in the extrauterine environment.^22^ This is not an easy task and may require longitudinal ultrasound measurements on a weekly basis, including biparietal diameter, head circumference, abdominal circumference, abdominal transverse diameter, femur length, estimated fetal weight, and head circumference–abdominal circumference ratio plotted in nomograms to detect stunted fetal growth as early as possible. However, the decision to terminate pregnancy is best taken in the early stage of detection of brain-sparing before decompensation and microstructural brain damage occur (eg, by Doppler flow analysis of the middle cerebral artery).^21^^,^^30^, ^31^, ^32^, ^33^ Conversely, if growth restriction and brain-sparing can be excluded (eg, in constitutionally small infants of small mothers, as in 146/213 [68.5%] cases in our sample [Table 5]), pregnancy should be optimally prolonged by all means to allow for further maturation of the brain and improved development during intrauterine growth.

As expected, EPH (edema, proteinuria, and hypertension) syndrome, hypertension during pregnancy, and multiple pregnancy causing placental insufficiency and pathologic heart rate patterns were associated with poor pO2, increased pCO2, and low pH in umbilical arterial blood in growth-restricted newborns (Tables 1 and 2). This evidence of chronic and/or acute hypoxemia reminds us to apply prospective risk management in clinical obstetrics and early intervention to prevent or ameliorate harm to growth-restricted infants (eg, by timely detection of pathologic cardiotocography patterns that are significantly more frequent in infants with IUGR than in SGA infants without growth restriction [62% vs 38%; *P*=.002]).^3^^,^^22^

### Strengths and limitations

This study demonstrates the differential effects of both growth restriction and immaturity on *predicted* psychomotor development at the preschool age of 4.3 (SD, 0.8) years in a prospective cohort of 854 preterm infants born at ≤37^+6/7^ weeks from a large ultrasound screening trial (n=5,301). This made possible the important observation that the specific detrimental effects of growth restriction on the fetal brain as compared with immaturity appear unrelated to cerebral hemorrhage and/or white matter damage. Hence, these results predicting psychomotor development at preschool age in risk groups are significant for clinical management of growth restriction and immaturity and parental consultation as well as for developing early intervention strategies to improve preschool support and reduce psychopathology in children. A general limitation of this study is that the preterm infants were part of a prospective cranial ultrasound screening performed from 1984 to 1988. Although this holds true for growth-restricted infants and for immature infants, this database is a valid source for the prediction of psychomotor trajectories among preschool-aged children within the boundaries of the data collection period. Furthermore, unlike information on IUGR, precise information on genetically or metabolically small infants in the SGA group is not provided in the screening database, and no cerebral Doppler studies are available.

### Conclusion

Compared with immaturity, growth restriction in preterm infants born at ≤37^+6/7^ weeks of gestation has more intense detrimental effects on *predicted* psychomotor development that increase with decreasing gestational age. Hence, this study demands for risk stratification of growth restriction and immaturity in preterm infants, thus enhancing clinical management, parental consultation, and the orchestration of early intervention strategies to improve preschool support, educational success, and mental health in children. The mechanisms of brain injury specific to growth restriction in preterm infants require further elucidation.

## CRediT authorship contribution statement

**Arne Jensen:** Data curation, Writing – original draft, Writing – review & editing. **Niels Rochow:** Conceptualization. **Manfred Voigt:** Conceptualization. **Gerhard Neuhäuser:** Conceptualization, Investigation, Writing – review & editing.
